# Higher levels of systemic immune-inflammatory index are associated with the prevalence of gallstones in people under 50 years of age in the United States: a cross-sectional analysis based on NHANES

**DOI:** 10.3389/fmed.2023.1320735

**Published:** 2024-01-12

**Authors:** Chunli Meng, Kai Liu

**Affiliations:** Center of Infectious Diseases, West China Hospital, Sichuan University, Chengdu, China

**Keywords:** gallstones, systemic immune-inflammatory index, SII, cross-sectional study, neutrophil, lymphocyte

## Abstract

**Introduction:**

Inflammation plays a significant role in gallstones formation. The prognosis of various illnesses was initially predicted using the systemic immune-inflammatory index (SII). We performed an updated analysis on the impact of SII and gallstones.

**Methods:**

To investigate the connection between the SII and gallstones occurrence in a sample of individuals from the National Health and Nutrition Examination Survey (NHANES) database from 2017 to 2020, we employed logistic regression analysis, subgroup analysis, and smoothing curve fitting.

**Results:**

In our study, an aggregate of 4,950 individuals over the age of 20 were enrolled, and 429 of them claimed to have gallstones. A fully adjusted model showed that the third and fourth quartiles of SII was parallel associated with gallstones in adults (OR = 2.43, 95% CI = 1.39–4.26; OR = 2.97, 95% CI = 1.72–5.16) under 50 years. Subgroup analysis and smoothed curve fitting provided evidence in favor of this finding.

**Conclusion:**

According to our research, gallstones are more likely to occur in US adults younger than 50 years.

## 1 Introduction

Gallstone disease is one of the most common digestive disorders and a significant factor in gastrointestinal hospitalization (1, 2). Gallstones are more frequently found in geriatric people and in women than in young people and men (3). Studies of the natural course of the disease have shown that the annual incidence of gallstones is 0.60–1.39% (4). Gallstones are now the second most common primary diagnosis for all gastrointestinal, liver, and pancreatic disorders in the United States, with a prevalence of approximately 20% in developed countries, which is significantly greater than that in developing countries, and the incidence of gallstones continues to increase (5–7). This poses a significant healthcare burden in the United States. Gallstones typically do not cause symptoms, but 3–8% of patients experience serious complications, such as acute cholecystitis, gallstone intestinal obstruction, pancreatitis, sepsis or perforation of the gallbladder (2, 8–10). For these reasons, gallstone disease is acknowledged as a significant public health issue.

Gallstones are solid biliary conglomerates consisting of calcium bilirubinate, mucus, cholesterol monohydrate crystals, and protein aggregates and can be categorized into cholesterol gallstones and pigment gallstones, with the former accounting for more than 70% of all gallstones (5, 11). Age, female, race/genetics, pregnancy, family history of gallstones, and sedentary lifestyle are a few well-known risk factors for gallstones (1, 12–14).

Inflammation is a key factor in the formation of gallstones according to recent studies. There is a substantial association between circulating inflammatory indicators and inflammatory proteins detected in bile according to studies examining the relationship between inflammation and the likelihood of developing gallstones (15). Additionally, both high-sensitivity C-reactive protein (hs-CRP) and C-reactive protein (CRP) are significantly linked to a graeter risk of gallstone disease (16, 17). Gallstones caused by cholesterol have also been linked to certain inflammatory illnesses, such as *Helicobacter pylori* infection (18). Researchers have also discovered that combining peripheral lymphocyte, neutrophil, and platelet counts may be a more accurate predictor of the inflammatory state, which is a sign of many diseases (19). Initially, used as a prognostic indicator for conditions such as cancer, cerebral hemorrhage, and coronary artery stenosis, the systemic immune-inflammatory index (SII) was first discovered (20, 21). However, nothing is known about the ability of the SII to predict gallstones, and its impact on gallstones has not yet been thoroughly understood. We proposed that SII is a predictor of the risk of gallstones. The present study investigated the association between the SII and gallstones.

## 2 Materials and methods

### 2.1 Data source and study population

**Figure 1:**
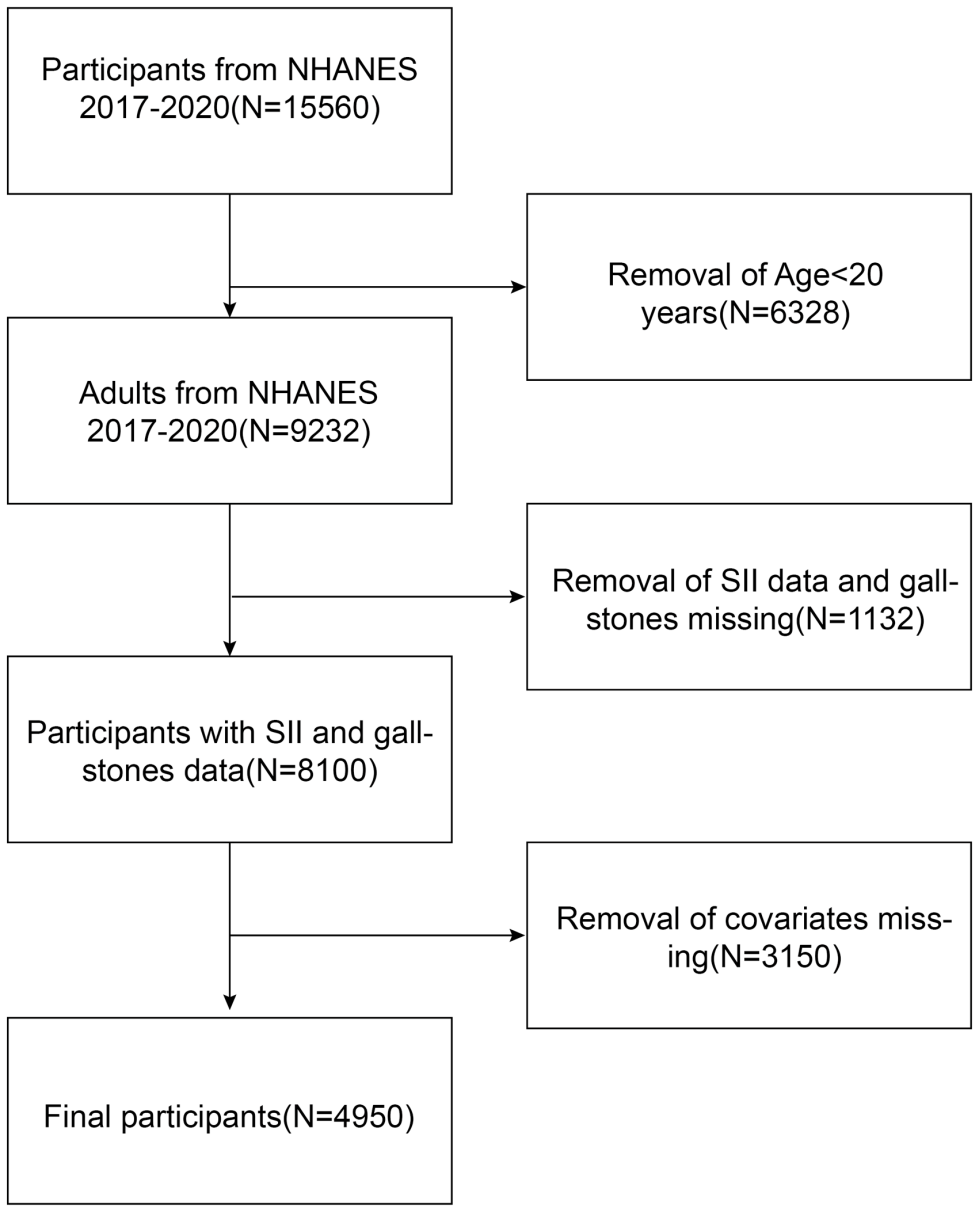
Flowchart for choosing participants. National Health and Nutrition Examination Survey, or NHANES.

The National Center for Health Statistics (NCHS) carried out the National Health and Nutrition Examination Survey (NHANES), a national survey with a complex, multistage design. The survey data were released every 2 years. The NCHS Ethics Review Board approved our cross-sectional survey study, and information about the data and study design can be obtained online at https://www.cdc.gov/nchs/nhanes/. Prior to collecting demographic, dietary, screening, laboratory, and questionnaire data, each survey respondent provided informed consent (22, 23). A total of 15,560 individuals signed up for the NHANES 2017–2020 survey. After cleaning the data, we eliminated those under the age of 20, missing covariate data, information about the SII, and information about gallstones. Finally, 4,950 US adults were included in our dataset for analysis. Sample selection was carried out as shown in Figure 1.

### 2.2 Definition of the SII and gallstones

Automated hematology analytical equipment (Coulter DxH 800 analyzer) was used to determine neutrophil, lymphocyte, and platelet counts, which were calculated as 10^®^3 cells/l. The SII was defined as the platelet count × neutrophil count/lymphocyte count. Gallstone history was defined by “Has DR ever said you have gallstones?.” The participants who responded to the inquiry had a history of gallstones that had been medically identified (20, 21).

### 2.3 Assessment of covariates

This study included an assortment of covariates based on the literature (1, 2, 12, 15), such as age, sex (male, female), household poverty-to-income ratio (PIR), educational level (below high school, high school, and above), smoking history (never, former, current), body mass index (BMI), cholesterol level (mg/dl), diabetes and cardiovascular disease (CVD), and dietary intake factors Between 2017 and 2020, every person performed a 24 h dietary recall; our analyses used the initial recall rate. BMI was divided into two categories: nonobese (<25 kg/m^2^) and obese (≥25 kg/m^2^). A history of CVD was defined as having received a diagnosis of coronary heart disease, congestive heart failure, a heart attack, a stroke, or angina. A self-reported diagnosis of diabetes, self-reported use of diabetes medications, or a fasting blood glucose level > 126 mg/dL or an HbA1c level ≥ 6.5% were the criteria for the diagnosis of diabetes.

The study also utilized participant self-report questionnaire data to identify variables such as smoking and drinking habits.

### 2.4 Statistical analysis

For continuous variables, baseline demographic characteristics are described as the means and standard deviations and for categorical variables, they are described as weighted percentages.

The connection between the SII and gallstones was examined by multifactor logistic regression analysis. Covariates in Model 1 were not adjusted. Gender, age, race/ethnicity, the household income to poverty ratio, and education level were all taken into account in Model 2. Model 3 had additional adjustments to account for BMI, smoking and drinking history, diabetes mellitus (DM) status, and food intake parameters (such as caloric, fat, sugar, and water intake). We carried out smoothed curve fitting and subgroup analysis to further investigate the connection between the SII and gallstones.

The statistical analyses were carried out using EmpowerStats software^1^ and the R package 3.6.1.^2^ *P* value of 0.05 were considered to indicate statistical significance.

## 3 Results

### 3.1 Baseline characteristics

Table 1 lists the characteristics of the NHANES 2017–2020 participants stratified by the SII. This study included 4,950 adult participants in total. The average age was 48.23 ± 16.97 years, with 2,272 (49.50%) female and 2,318 (50.50%) male individuals. Significant associations between SII and different factors were found through this study. Participants in the high SII quartile typically had a higher BMI and were more likely to be female, non-Hispanic White, or Gallstones patients.

**Table 1:** Characteristics of the study population based on SII groups in NHANES 2017–2020 (*n* = 4,950).

SII groups	Q1 (≤314.9)	Q2 (314.9–446.9)	Q3 (446.9–608.7)	Q4 (>608.7)	*p*-value
N	1,147	1,148	1,147	1,148	
Age (years)	48.57 ± 16.40	47.65 ± 17.06	47.84 ± 17.05	48.85 ± 17.35	0.266
TC (mg/dl)	186.75 ± 41.37	188.35 ± 41.80	186.34 ± 39.58	186.04 ± 40.99	0.536
Energy (kcal)	2233.65 ± 1037.03	2204.29 ± 1042.23	2187.09 ± 987.35	2218.29 ± 1033.69	0.743
Total sugars (g)	104.76 ± 75.54	103.06 ± 73.06	107.74 ± 74.22	110.99 ± 80.91	0.073
Total fat (g)	90.61 ± 50.54	90.81 ± 52.14	88.98 ± 47.20	90.90 ± 50.42	0.784
Total moisture (g)	3049.70 ± 1622.86	2993.15 ± 1563.16	2990.14 ± 1633.23	3044.45 ± 1545.70	0.718
Gender					<0.001
Male	669 (58.33%)	601 (52.35%)	523 (45.60%)	525 (45.73%)	
Female	478 (41.67%)	547 (47.65%)	624 (54.40%)	623 (54.27%)	
BMI					<0.001
<25 (kg/m^2^)	335 (29.21%)	290 (25.26%)	255 (22.23%)	248 (21.60%)	
≥25 (kg/m^2^)	812 (70.79%)	858 (74.74%)	892 (77.77%)	900 (78.40%)	
Race					<0.001
Mexican American	132 (11.51%)	161 (14.02%)	142 (12.38%)	123 (10.71%)	
Other Hispanic	91 (7.93%)	121 (10.54%)	105 (9.15%)	132 (11.50%)	
Non-Hispanic White	299 (26.07%)	448 (39.02%)	491 (42.81%)	533 (46.43%)	
Non-Hispanic Black	436 (38.01%)	267 (23.26%)	250 (21.80%)	196 (17.07%)	
Other race	189 (16.48%)	151 (13.15%)	159 (13.86%)	164 (14.29%)	
Educational level					0.727
Below high school	137 (11.94%)	158 (13.76%)	156 (13.60%)	158 (13.76%)	
High school	265 (23.10%)	259 (22.56%)	256 (22.32%)	276 (24.04%)	
Above	745 (64.95%)	731 (63.68%)	735 (64.08%)	714 (62.20%)	
PIR					0.052
<1.8	302 (26.33%)	268 (23.34%)	274 (23.89%)	297 (25.87%)	
1.8–2.3	430 (37.49%)	429 (37.37%)	433 (37.75%)	470 (40.94%)	
>2.3	415 (36.18%)	451 (39.29%)	440 (38.36%)	381 (33.19%)	
Alcohol					0.369
≤2 drink/day	946 (82.48%)	914 (79.62%)	932 (81.26%)	927 (80.75%)	
>2 drink/day	201 (17.52%)	234 (20.38%)	215 (18.74%)	221 (19.25%)	
Smoke status					0.499
Never	641 (55.88%)	639 (55.66%)	643 (56.06%)	610 (53.14%)	
Former	279 (24.32%)	290 (25.26%)	282 (24.59%)	281 (24.48%)	
Now	227 (19.79%)	219 (19.08%)	222 (19.35%)	257 (22.39%)	
Diabetes					0.290
Yes	182 (15.87%)	186 (16.20%)	195 (17.00%)	214 (18.64%)	
No	965 (84.13%)	962 (83.80%)	952 (83.00%)	934 (81.36%)	
Gallstones					0.019
Yes	86 (7.50%)	99 (8.62%)	119 (10.37%)	125 (10.89%)	
No	1,061 (92.50%)	1,049 (91.38%)	1,028 (89.63%)	1,023 (89.11%)	
CVD					0.301
Yes	39 (3.40%)	35 (3.05%)	51 (4.45%)	39 (3.40%)	
No	1,108 (96.60%)	1,113 (96.95%)	1,096 (95.55%)	1,109 (96.60%)	

TC, Total cholesterol; PIR, Poverty-to-income ratio; BMI, Body mass index; CVD, Cardiovascular disease.

Mean ± SD for Age (years), Total cholesterol (mg/dl), Energy (kcal), Total sugars (g), Total fat (g), Total moisture (g); *p* value was calculated by the weighted linear regression model. % for: Gender, Race, Educational level, PIR, Gallstones, Smoking status, Alcohol, CVD and Diabetes. *p* value was calculated by the weighted chi-square test.

### 3.2 Association between the SII and gallstones

The relationship between the SII and gallstones is shown in Table 2. A higher SII (third quartile and the highest quartile) was significantly linked to a greater likelihood of developing gallstones than as the first quartile (Q1) according to the unadjusted model (OR = 1.43, 95%CI = 1.07–1.91, *p* = 0.016; OR = 1.51, 95% CI = 1.13–2.01, *p* = 0.005). Model I with covariate adjustments for sex, age, race, education level, and PIR did not show any statistically significant differences, and Model II, adjusted for additional covariates, also showed no statistically significant differences. However, stratified analyses showed (Table 3) that after adjusting for all covariates, the SII in the highest quartile (Q4) was strongly associated with gallstones among adults <50 years (OR = 2.97, 95%CI = 1.72–5.16, *p* = 0.0001); BMI ≥25 kg/m^2^ (OR = 1.44, 95%CI = 1.09–1.99, *p* = 0.024); nonalcoholics (OR = 1.50, 95%CI = 1.09–2.06, *p* = 0.013); nondiabetics (OR = 1.13, 95%CI = 1.16–2.30, *p* = 0.005), and nosmokers (OR = 1.79, 95%CI = 1.18–2.73, *p* = 0.007). These findings imply that a variety of variables may have an impact on the relationship between the SII and the likelihood of gallstone prevalence.

**Table 2:** The associations between SII and gallstones.

SII	Non-adjusted		Adjust I		Adjust II	
	OR (95% CI)	*p*-value	OR (95% CI)	*p*-value	OR (95% CI)	*p*-value
Q1	1.0		1.0		1.0	
Q2	1.16 (0.86, 1.57)	0.322	1.07 (0.78, 1.46)	0.669	1.04 (0.75, 1.43)	0.821
Q3	1.43 (1.07, 1.91)	0.016	1.26 (0.93, 1.70)	0.140	1.08 (0.79, 1.48)	0.626
Q4	1.51 (1.13, 2.01)	0.005	1.29 (0.96, 1.75)	0.096	1.10 (0.80, 1.51)	0.554

The non-adjusted model adjusts for: None. Adjust I model to adjust for Gender; Age (years); Race; Ratio of family income to poverty; Education; Adjust II models adjust for Gender; Age (years); Race; Ratio of family income to poverty; Education; BMI; Energy (kcal); Total sugars (g), Total fat (g), Total moisture (g); Drinking; Smoking; Diabetes and Total cholesterol.

**Table 3:** Stratified analyses for the association between SII and gallstones.

Stratified variable	N	Q1	Q2	Q3	Q4
		Ref	OR (95% CI) *p*-value	OR (95% CI) *p*-value	OR (95% CI) *p*-value
Gender					
Male	2,318	1.0	1.50 (0.90, 2.49)	0.95 (0.53, 1.70)	1.58 (0.94, 2.66)
			0.120	0.857	0.084
Female	2,272	1.0	0.98 (0.66, 1.45)	1.31 (0.91, 1.88)	1.22 (0.84, 1.75)
			0.921	0.148	0.293
Age (years)					
<50	2,420	1.0	1.64 (0.91, 2.98)	2.43 (1.39, 4.26)	2.97 (1.72, 5.16)
			0.101	0.002	0.0001
≥50	2,170	1.0	1.12 (0.77, 1.62)	1.10 (0.76, 1.59)	0.99 (0.68, 1.44)
			0.545	0.618	0.974
BMI					
<25 (kg/m^2^)	1,128	1.0	1.09 (0.48, 2.45)	0.79 (0.32, 1.95)	0.94 (0.39, 2.27)
			0.838	0.606	0.890
≥25 (kg/m^2^)	3,462	1.0	1.16 (0.83, 1.62)	1.38 (1.00, 1.90)	1.44 (1.05, 1.99)
			0.386	0.053	0.024
Race					
Mexican American	558	1.0	0.75 (0.33, 1.68)	0.73 (0.32, 1.67)	0.82 (0.36, 1.90)
			0.482	0.455	0.649
Other Hispanic	449	1.0	1.80 (0.69, 4.68)	1.46 (0.53, 4.03)	1.06 (0.39, 2.91)
			0.227	0.468	0.907
Non-Hispanic White	1771	1.0	0.61 (0.36, 1.05)	1.19 (0.75, 1.91)	1.24 (0.78, 1.97)
			0.074	0.461	0.353
Non-Hispanic Black	1,149	1.0	1.98 (1.06, 3.70)	1.78 (0.93, 3.43)	1.59 (0.78, 3.24)
			0.033	0.082	0.202
Other Race	663	1.0	1.56 (0.69, 3.54)	0.99 (0.41, 2.40)	1.36 (0.59, 3.12)
			0.289	0.983	0.475
Alcohol					
≤2 drink/day	3,719	1.0	1.19 (0.85, 1.67)	1.36 (0.98, 1.88)	1.50 (1.09, 2.06)
			0.300	0.063	0.013
>2 drink/day	871	1.0	1.26 (0.57, 2.79)	1.45 (0.65, 3.24)	1.28 (0.57, 2.90)
			0.569	0.365	0.546
Smoke status					
Never	2,533	1.0	1.11 (0.70, 1.75)	1.44 (0.93, 2.23)	1.79 (1.18, 2.73)
			0.652	0.100	0.007
Former	1,132	1.0	1.08 (0.63, 1.85)	1.17 (0.69, 2.00)	1.14 (0.67, 1.95)
			0.784	0.553	0.627
Now	925	1.0	1.52 (0.78, 2.99)	1.43 (0.73, 2.81)	1.12 (0.56, 2.22)
			0.222	0.294	0.754
Diabetes					
Yes	777	1.0	1.67 (0.93, 2.98)	1.32 (0.73, 2.38)	0.89 (0.48, 1.63)
			0.085	0.360	0.707
No	3,813	1.0	1.03 (0.71, 1.49)	1.35 (0.95, 1.92)	1.63 (1.16, 2.30)
			0.873	0.092	0.005

The results of stratified analysis were adjusted for Gender; Age (years); Race; Ratio of family income to poverty; Education; BMI; Energy (kcal); Total sugars (g), Total fat (g), Total moisture (g); Drinking; Smoking; Diabetes and Total cholesterol; 95% CI, 95% Confidence Interval; OR, Odds Ratio; BMI was categorized as no-obese (<25 kg/m^2^), and obese (≥25 kg/m^2^).

Table 4 shows the results of a subgroup analysis that examined the correlation between SII score and gallstone risk, including of sex, age, race, BMI, smoking status, alcohol consumption, and diabetes. The SII and gallstones significantly correlated in adults under 50 year (OR = 1.001, 95%CI = 1.000–1.001, *p* = 0.0001). A significant interaction effect between the SII and BMI or nondiabetic patients was also found (*p* < 0.05). The SII was significantly associated with an increased incidence of gallstones in participants with a BMI ≥ of 25 kg/m^2^ (OR = 1.000, 95%CI = 1.000, 1.001, *p* = 0.014) and nondiabetic participants (OR = 1.000, 95%CI = 1.000, 1.001, *p* = 0.002).

**Table 4:** A subgroup of analyses for the association between SII and gallstones.

Subgroup	N	OR (95% CI)	*p* value	p (interaction)
Gender				0.504
Male	2,318	1.000 (1.000, 1.001)	0.393	
Female	2,272	1.000 (1.000, 1.001)	0.101	
Age (years)				0.029
<50	2,420	1.001 (1.000, 1.001)	0.0001	
≥50	2,170	1.000 (1.000, 1.000)	0.746	
BMI				0.136
<25 (kg/m^2^)	1,128	1.000 (0.999, 1.001)	0.662	
≥25 (kg/m^2^)	3,462	1.000 (1.000, 1.001)	0.014	
Race				0.462
Mexican American	559	1.000 (0.999, 1.001)	0.620	
Other Hispanic	449	1.000 (0.999, 1.001)	0.829	
Non-Hispanic White	1,171	1.000 (1.000, 1.001)	0.374	
Non-Hispanic Black	1,149	1.001 (1.000, 1.001)	0.024	
Other Race	663	1.000 (0.999, 1.001)	0.704	
Alcohol				0.334
≤2 drink/day	3,719	1.000 (1.000, 1.001)	0.012	
>2 drink/day	871	1.000 (0.999, 1.001)	0.893	
Smoke status				0.722
Never	2,533	1.000 (1.000, 1.001)	0.050	
Former	1,132	1.000 (1.000, 1.001)	0.522	
Now	925	1.000 (1.000, 1.001)	0.414	
Diabetes				0.002
Yes	777	1.000 (0.999, 1.000)	0.170	
No	3,813	1.000 (1.000, 1.001)	0.002	

The results of subgroup analysis were adjusted for Ratio of family income to poverty; Education; BMI; Energy (kcal); Total sugars (g), Total fat (g), Total moisture (g) and Total cholesterol.95% CI, 95% Confidence Interval; OR, Odds Ratio; BMI was categorized as no-obese (<25 kg/m^2^), and obese (≥25 kg/m^2^).

**Figure 2:**
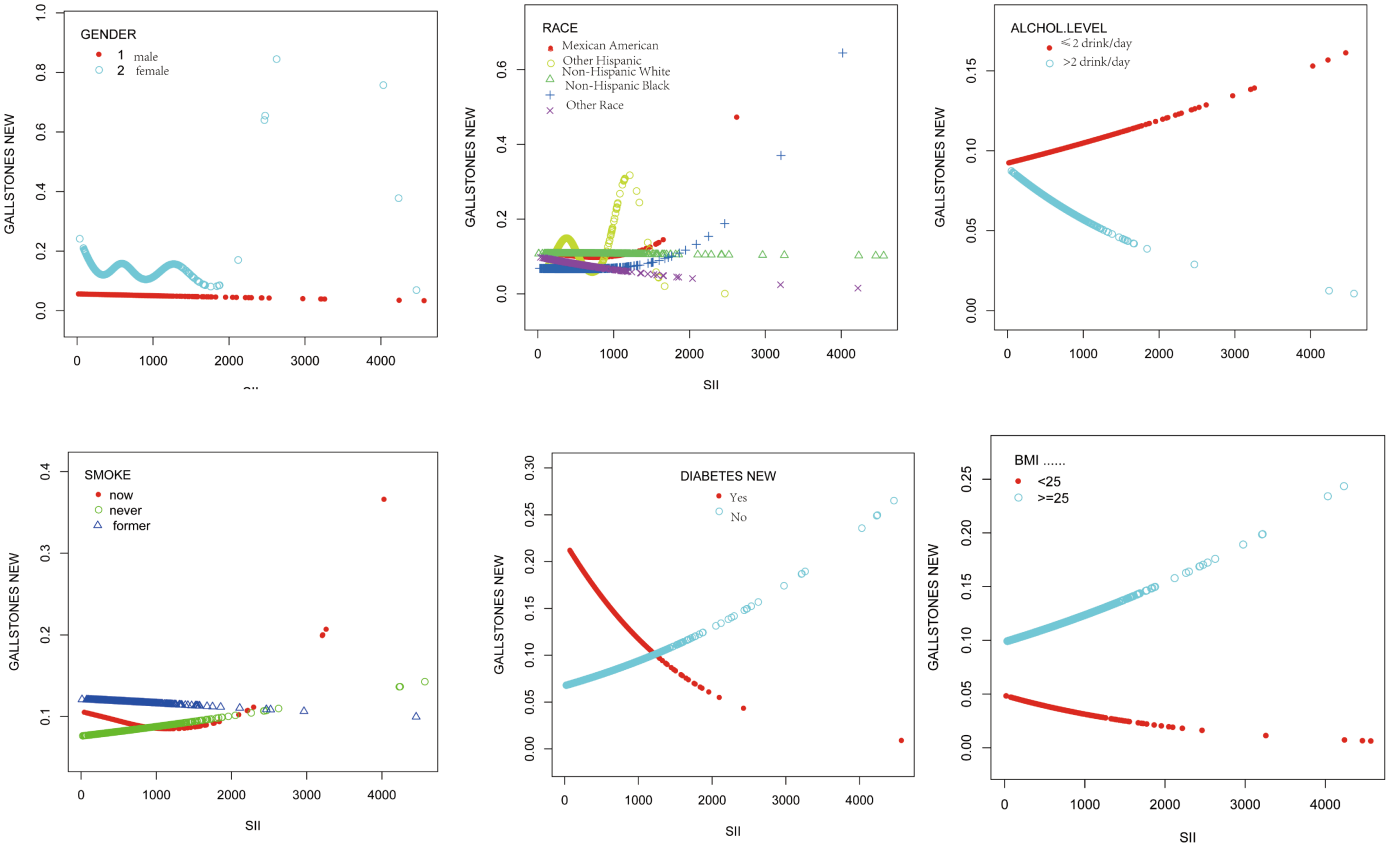
The associations between SII and gallstones were stratified by sex, age, ethnicity, BMI, smoking, alcohol consumption, and diabetes.

Smoothed curve fitting results stratified by sex, age, ethnicity, BMI, smoking status, alcohol consumption status, and diabetes status showed that the SII was positively associated with the likelihood of gallstone in participants under 50 years and with a BMI ≥25 kg/m^2^ (Figure 2).

## 4 Discussion

This study evaluated the association SII and gallstones of individuals from 4,950 US individuals from the NHANES 2017–2020 database. The immune system has long been recognized as a prognostic factor for a number of diseases. A higher SII score was shown to be independently linked to a risk of gallstones in individuals under 50 years in this cross-sectional investigation. Even after potential confounders, including sex, age, race/ethnicity, and PIR etc., the association was still maintained. This conclusion was validated by subgroup analysis and smoothed curve fitting. In addition, as the SII score increased, the risk of gallstones was greater in obese and nondiabetic individuals. Importantly, the measurement of the SII is easy to access, which is based on widely used laboratory techniques for determining platelet, lymphocytes, and peripheral neutrophil counts in clinical practice. The SII may be used as a biomarker for gallstones in young people for the purposes of this article.

Previous studies have revealed that inflammation is an important contributor to the occurrence of gallstones, and the immune inflammatory response has been linked to a number of disease processes (2, 16). In one of these investigations, Liu et al. examined into the connection between circulating inflammatory proteins and gallstones and discovered that four ILs—IL-6, IL-10 etc.—were linked to a greater incidence of gallstones (15). C-reactive protein (CRP) and gallstones are directly correlated, with an OR of 1.03, according to Shabanzadeh et al.’s study on the connection between metabolic biomarkers and gallstones (24). High-sensitivity C-reactive protein (hs-CRP) concentrations were found to be strongly related to an increased risk of gallstones in a study by Tong Liu (16). Inflammation is a crucial component in the production of cholesterol gallstones, according to research on a prairie dog model of gallstone formation. In particular, gallstones was detected when high dosages of acetylsalicylic acid (aspirin) were fed to prairie dogs, yet gallstones were not detected in control animals (25, 26).

Studies using mature T or B-cell-free animals (Rag mice), which lack these cells, provide additional proof of the involvement of the immune system in the etiology of gallstones. Only wild-type mice were found to have a high frequency of gallstones (26, 27). This implies that the adaptive immune system, namely, T-cell activity, is likely to be activated by solid cholesterol crystals to cause inflammation. A significant body of research has been performed on the prognostic value of the SII as a straightforward, trustworthy, and less intrusive biomarker in a variety of illnesses. Since lymphocytes are a subgroup of leukocytes that regulate innate and adaptive immune responses, lower peripheral lymphocyte numbers correspond to greater SII values. Additionally, platelets are becoming increasingly acknowledged as important regulators of the inflammatory response. An intrinsic coagulation cascade that results from activated platelets can cause a number of illnesses. Inflammatory conditions can potentially be accelerated by platelets. Monocytes, neutrophils, and lymphocytes contact platelets, which helps to control innate and adaptive responses (19, 28).

Gallstones are the result of a synergistic interaction between hereditary and environmental factors (26). Female, obesity, and diabetes are risk factors for gallstones (1). It is debatable whether age has a role in gallstone development. In the present study, our findings demonstrated the association of the SII with gallstones only in people under 50 years and in obese individuals. In fact, older people have more risk factors than younger people, such as obesity and metabolic syndrome, which are risk factors for diseases in older people; therefore, the effect of the SII on gallstones may be masked by other factors. In addition, the study was shown that the SII is associated with a greater risk of gallstones in nondiabetic patients. However, in this study, diabetes was not specifically typed. Chia-Hung Kao et al. reported a strong correlation between type 2 diabetes and gallstones, but there was a negative correlation between the prevalence of type 1 diabetes and gallstones in patients aged 20–40 years (29). However, in a cohort by Torben Jørgensen et al., gallstone disease was found to be associated with the development of any autoimmune disease, driven mainly by type 1 diabetes and autoimmune thyroid disease. Therefore, the relationship between gallstones and diabetes is currently unclear, and additional studies are needed to confirm it (30). It is worth proposing that although high SII scores were found to be significantly associated with gallstones risk in the nondiabetic population in this study, inflammation leads to insulin resistance. Perhaps this group of participants were not diabetic but actually had insulin resistance during inflammation. And previous studies (31) have indicated that insulin resistance predisposes patients to bile supersaturation by reducing bile salt secretion leading to bile supersaturation and increasing mucus production by inducing gallbladder inflammation. This is a possible reason why people who are non-diabetic but have a high SII index are susceptible to gallstones.

The accuracy of transabdominal ultrasound in detecting gallstones is more than 95% (32). In this study, high SII score was found to be positively associated with gallstones risk in people under 50 years of age. Therefore, transabdominal ultrasound testing is necessary to screen for gallstones in people under 50 years of age with high SII.

Our study provides an array of advantages that support the accuracy and precision of our findings. First, the NHANES participants were a representative sample of Americans who followed a carefully planned study protocol with stringent quality control and assurance to guarantee the accuracy of our results. To ensure that our findings held true for a wider variety of people, we also performed subgroup analyses and corrected for confounding variables. However, our study has several limitations. First, because it was a cross-sectional study, we were unable to determine how the SII and gallstones are causally related. Second, the entire gallstone dataset was derived through questionnaires, which could be biased by recall. Despite these drawbacks, the association between the SII and the occurrence of gallstones was initially revealed in this paper.

## 5 Conclusion

This cross-sectional study suggested that the SII was positively associated with gallstones in US adults aged less than 50 years and was more pronounced in the obese population. These findings complement those of previous studies, which still need additional large-scale prospective cohorts for validation.

## Data availability statement

The datasets presented in this study can be found in online repositories. The names of the repository/repositories and accession number(s) can be found in the article/supplementary material.

## Ethics statement

The studies involving humans were approved by The NCHS Ethics Review Board approved our cross-sectional survey study, and information about the data and study design can be obtained online at https://www.cdc.gov/nchs/nhanes/. The studies were conducted in accordance with the local legislation and institutional requirements. The participants provided their written informed consent to participate in this study.

## Author contributions

CM: Conceptualization, Data curation, Formal analysis, Methodology, Software, Writing – original draft. KL: Conceptualization, Data curation, Writing – review & editing.

## Footnotes

^1^ http://www.empowerstats.com

^2^ http://www.R-project.org
